# Diagnostic Reasoning in Internal Medicine. Cynefin Framework Makes Sense of Clinical Complexity

**DOI:** 10.3389/fmed.2021.641093

**Published:** 2021-04-22

**Authors:** Gino Roberto Corazza, Marco Vincenzo Lenti

**Affiliations:** Department of Internal Medicine, San Matteo Hospital Foundation, University of Pavia, Pavia, Italy

**Keywords:** aging, clinical complexity, conceptual framework, decision-making, multimorbidity

As a result of the continuous demographic changes, internal medicine is increasingly dealing with elderly patients suffering from chronic—and often multiple—diseases, and it is therefore the medical specialty in the closest relationship with clinical complexity (1).

Although in medicine a univocal definition of complexity has not been agreed so far (2), a complex system can be provisionally described as a network of individual factors from whose dynamic interaction new properties of the system itself emerge (3). Its most qualifying feature is represented by interconnectedness, and other essential features are non-linearity, unpredictability, adaptability, coevolution, and context sensitivity (4). It is now accepted that clinical complexity is something more and different from multimorbidity (5), and a recent consensus document (6) has identified a series of relevant determinants of complexity, either inherent (i.e., biological, such as aging, multimorbidity, frailty, disease severity, resilience) or contextual (i.e., non-biological, such as socioeconomic, cultural, environmental, behavioral factors) to the patient.

For many years, the best-known graphic conceptualization of complexity has been the Stacey diagram (7). It was conceived in the financial field, and it distinguishes an area of rationality, ruled by the usual mechanistic relationships of cause/effect, an area of chaos characterized by afinalistic turbulence and the interruption of normal connections, and a large intermediate area, that of complexity. It is within this latter, characterized by low levels of certainty and agreement, hence not evidence-based, that a large part of our complex patients fit in. This is the case, for example, of older patients with geriatric syndromes, who often suffer from frailty, cognitive impairment, higher fall risk and fall-related injuries, motor impairment, and poor health outcomes (e.g., hospital admission, mortality) (8, 9).

An additional evolution in the field of “knowledge management” is the so-called Cynefin framework (CF), which emerged from research conducted in the field of complex adaptive system theory developed by Snowden for decision-making in economics (10). As already happened for the Stacey diagram, it soon became clear that CF could prove useful for making sense of complexity even in the biomedical field (11–13). In fact, the CF provides a reference language to dynamically contextualize situations located in different domains (contexts) that require distinguished responses and characterized by different patterns of cause–effect relationship. More in depth, the CF was described as a “sense-making” tool, able to provide a reasoning pathway rather than a solution to a specific problem (10). Figure 1 shows how these four domains, which only partially overlap with the areas of the Stacey diagram, are embedded to each other. The simple domain is the only legitimate space of the best practice in which cause and effect are in a linear relation and easy to understand. Inside this area, obvious troubles and straightforward clinical problems can be solved through validated protocols, without the need for specialized medical knowledge and for further analysis or experimenting. In the complicated domain, only a good practice is possible by experts or focused physicians capable of grasping, by analytical tools and specialized knowledge, apparently hidden causalities and relationships which can, however, still be recognized. The complex domain is a fluid space of varying stabilities over time and space, and this is the case, for example, of patients with multiple chronic conditions, presenting with multifaceted clinical complaints, with complex social circumstances. The most correct attitude toward biological and non-biological factors is to abandon a reductionist mindset, and to reason in terms of systems and elements in continuous interaction (14), in order to probe the emergent causality and to get insight for a proper diagnosis. A cause–effect relationship can only be unveiled in retrospect, as no physician would immediately know *a priori* which solution is best, and this represents an opportunity for emergent practice. The chaotic domain is the space of medical urgencies or late-stage or novel diseases against which an expeditious and often innovative action is required to steer the system and stabilize the situation without targeting insight and decision making. An emblematic example is that of the severe acute respiratory syndrome coronavirus 2 (SARS-CoV-2) pandemic, which has put a lot of pressure at multiple levels, including the healthcare and the economic and political systems, also representing an unprecedented challenge for the whole scientific community (15). In this case, the fact that very little knowledge was available at first strongly favored innovative action that was needed to face the initial phases of the pandemic, especially for understanding SARS-CoV-2 infection clinical characteristics and outcomes. Table 1 reports four different clinical scenarios according to the four Cynefin domains. Indeed, these four domains should not be considered as “waterproof” compartments. If it is true that different domains make use of different thinking strategies, in reality they communicate through the circular flow of knowledge that is gradually generated, and CF actually provides a context-driven and flexible approach to medical care (16).

**Figure 1:**
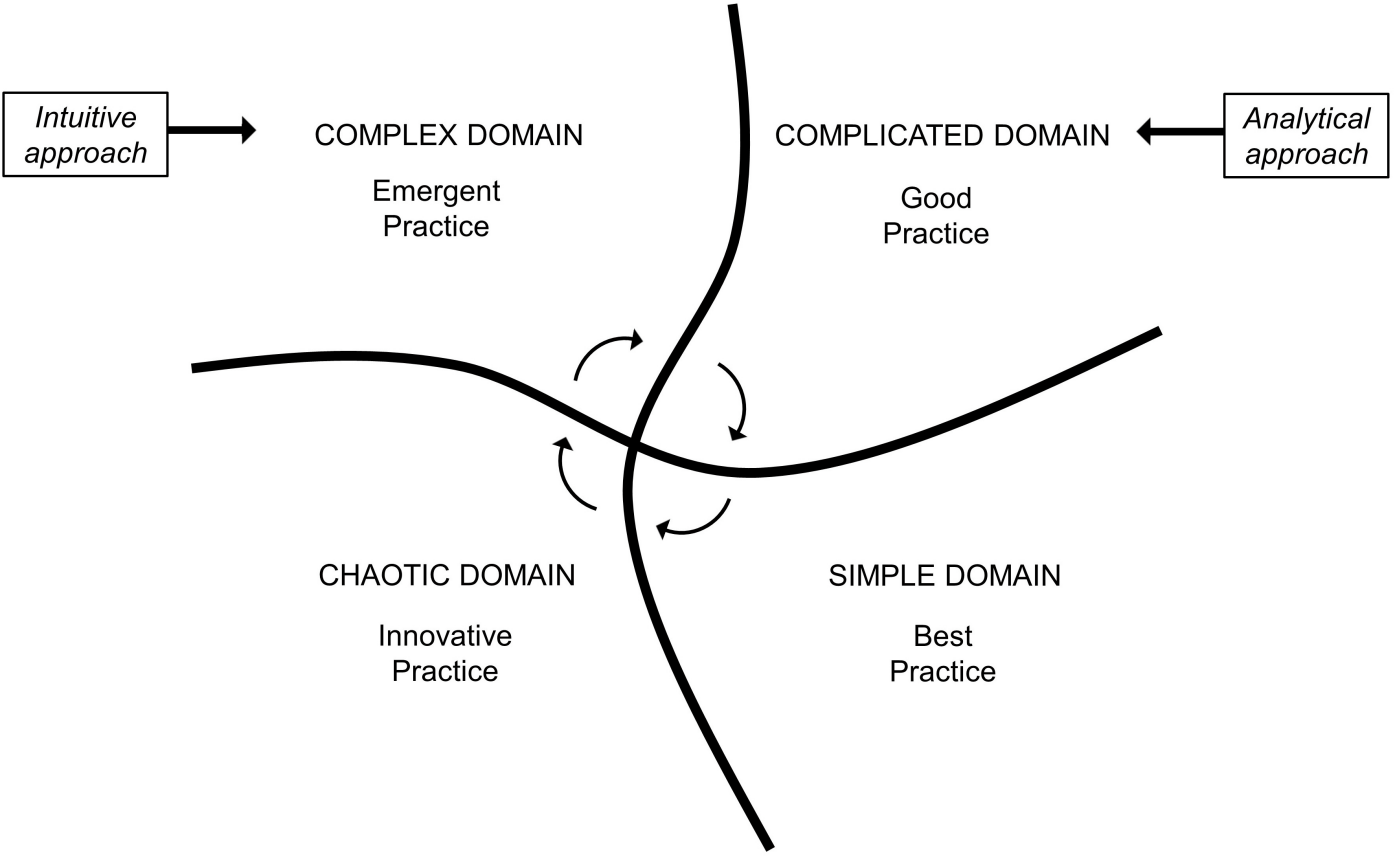
Cynefin identifies four different domains of medical knowledge. Each of them consents different standards of clinical practice and generates circular fluxes of knowledge. Different domains require different diagnostic approaches. Adapted and modified from Snowden & Boone (10).

**Table 1:** Examples of different clinical scenarios categorized according to the Cynefin framework.

**Cynefin domain**	**Clinical scenario**	**Reasoning strategy**
Simple	Minor cut on a finger causing negligible bleeding	Clear and simple cause–effect relationship. This scenario does not require specialist knowledge and the management is straightforward
Complicated	A 23-year-old woman presenting with abdominal pain, diarrhea, and tender red nodules on both shins	A cause–effect relationship can be found, but may not be immediately evident. Evaluate causes of erythema nodosum and abdominal pain; the analytical approach allows to identify the causality between the skin condition and a gastrointestinal disorder (Crohn's disease in this case)
Complex	A 78-year-old, socially deprived, man accessed the A&E for malaise, persisting fever, tachycardia, and hypotension; he has a history of chronic heart failure, essential hypertension, diverticulosis, and benign prostate hypertrophy; he lives alone and has no caregivers	A cause–effect relationship can only be unveiled in retrospect. After evaluation of laboratory tests, a preliminary diagnosis of decompensated diabetes mellitus and bacterial infection is made. Also, ulcers in the lower limbs are noticed. The patient has not been well for a long time, he had poor access to medical care, and he is not adherent to treatment plans. Reasoning in terms of interconnected systems is crucial in this case
Chaotic	A 62-year-old patient suffering from diabetes mellitus type 2, admitted to hospital for a car accident determining multiple injuries, including splenic rupture and hemorrhagic shock; the patient is unconscious	Cause–effect relationships cannot be found as they constantly evolve and change. In this case, a life-saving action comes first (urgent support), while other medical needs and testing prioritization are uncertain and depend on the outcome of resuscitation maneuvers; logic and rationality seem to fail in such a scenario

Having said that, the most important novelty of CF consists in having kept the boundaries between “complex” and “complicated.” Even in the scientific literature, the colloquial use of the term “complex” instead of “complicated” is not uncommon, but while the latter points to the general difficulty of understanding, the former implies the presence of interconnections that constitute the functional characteristic of the systems (17). In clinical practice, not realizing of being in the wrong context can have dire consequences. Due to the tendency to get an immediate control of a certain disease or condition, medicine usually treats many problems as complicated, thus requiring many tests and analyses (13) and ultimately getting overdiagnosis and resource waste (18). However, this tendency would not allow patterns to emerge, causing a final detrimental effect. This is truly a critical issue: complex problems cannot be addressed as the complicated sum of solvable subproblems, and, for facing them, an analytical and reductionist approach would dissolute those interactions that constitute their essence.

CF, representing a suitable conceptual platform to talk about clinical complexity, can contribute to better-oriented diagnostic processes. Internal medicine has a lot to do with the diagnostic processes (19) which are particularly susceptible to errors (20) due to the great dissimilarity among patients (21), as well as due to their individual complexity (4). Clinical diagnosis is a multistage process that takes advantage of a series of strategies (21, 22). Although maximum flexibility of thought has long been recommended, these strategies can essentially be traced back to an intuitive (23) and analytical (24) approach. The intuitive approach is based on the so-called tacit knowledge arising from a wealth of experience enriched by personal perceptions and beliefs which are hard to transfer. The analytic approach is based on an explicit knowledge based on facts, rules, and procedures codified in paper or electronic form and already widely accepted (25). This analytical approach consciously uses a hypothetical–deductive logic and is time- and resource-consuming. The intuitive approach, on the contrary, unconsciously uses short mental circuits through which the patient's clinical picture as a whole is automatically compared, according to a pattern recognition process to a prototype of disease which is already present in the physician's experiential memory.

Patients with rare diseases, with atypical presentation, with an unusual course or who require instrumental tests with advanced technology for being diagnosed, place themselves in the CF in a complicated context and require an analytical approach based on evidence-based algorithms managed by doctors with specialized training (Figure 1). The same reductionist approach is not as effective, and above all efficient, for the diagnosis of those complex patients who currently make up the vast majority in outpatient clinics and internal medicine wards. Older, fragile patients with multimorbidity and polytherapy, perhaps accompanied by abnormal behaviors and social distress, even if characterized by unusual disease course or atypical presentation (26), must be studied through an intuitive, multidimensional and holistic approach, which constitutes an essential qualifier of internal medicine and is crucial for the embracement of clinical complexity (27). This approach is faster and tends to consider contextual aspects and to avoid, as far as possible, expensive and invasive tests. In other terms, this represents an emergent practice, which is an action-oriented approach (28) that generates a new, patient-centered, opportunity. Indeed, the recognition of complex patients remains the most difficult challenge in clinical practice. Therefore, rather than with the use of a clinical guideline, patients can be categorized within the Cynefin framework by applying a score which quantifies clinical complexity. However, a universally accepted tool for assessing clinical complexity is yet to be developed (4).

Concerning the potential role of machine learning in aiding clinical decisions, this has been widely discussed (29, 30). In brief, machine learning is an algorithm that is able to learn by using a huge amount of data which certainly exceed the capacity of a human mind (29). However, if from a theoretical point of view this could potentially allow a more precise diagnosis, convincing evidence about its feasibility and usefulness is still lacking.

If CF does not offer concrete scientific or clinical solutions, it certainly represents a useful conceptual framework to capture the critical consequences of clinical complexity (12). In the day-by-day medical activity, it must be kept in mind that complex and complicated domains are different contexts, characterized by different clinical practice standards and that require different diagnostic reasoning approaches.

## Author Contributions

All authors participated in the drafting of the manuscript or critical revision of the manuscript for important intellectual content and provided approval of the final submitted version.

## Conflict of Interest

The authors declare that the research was conducted in the absence of any commercial or financial relationships that could be construed as a potential conflict of interest.

## Abbreviations

CF: cynefin framework.
